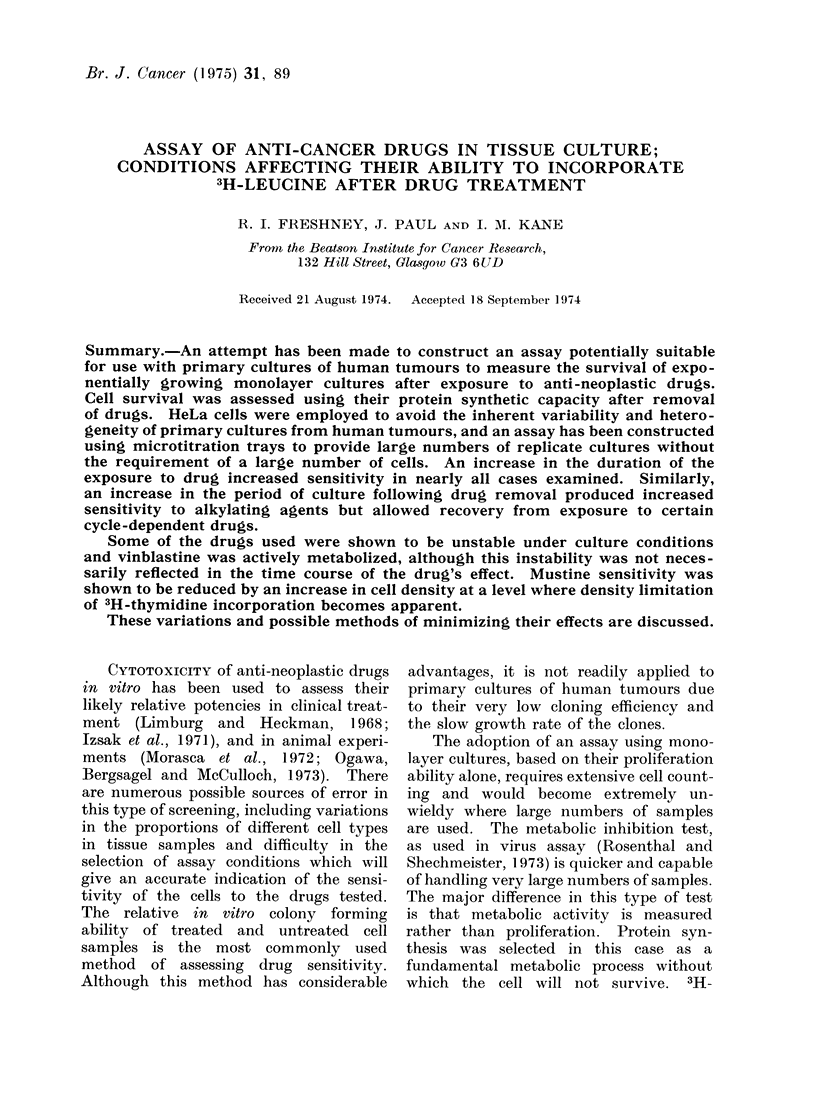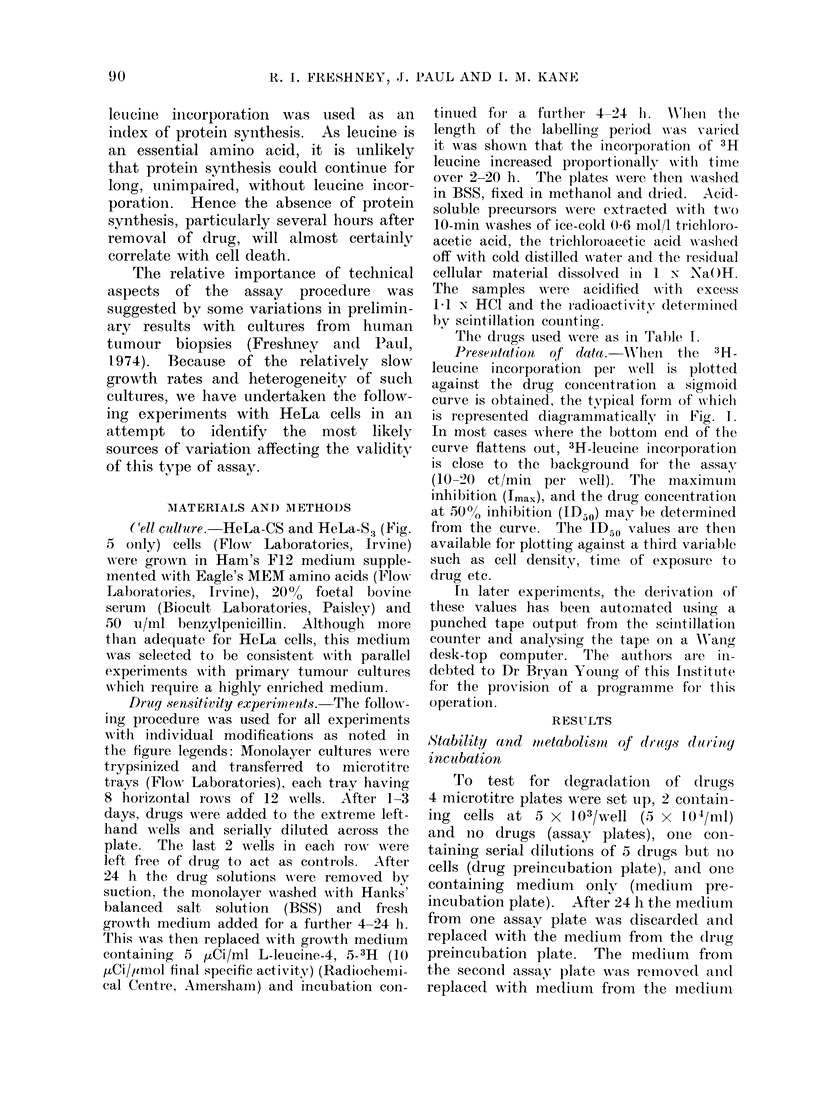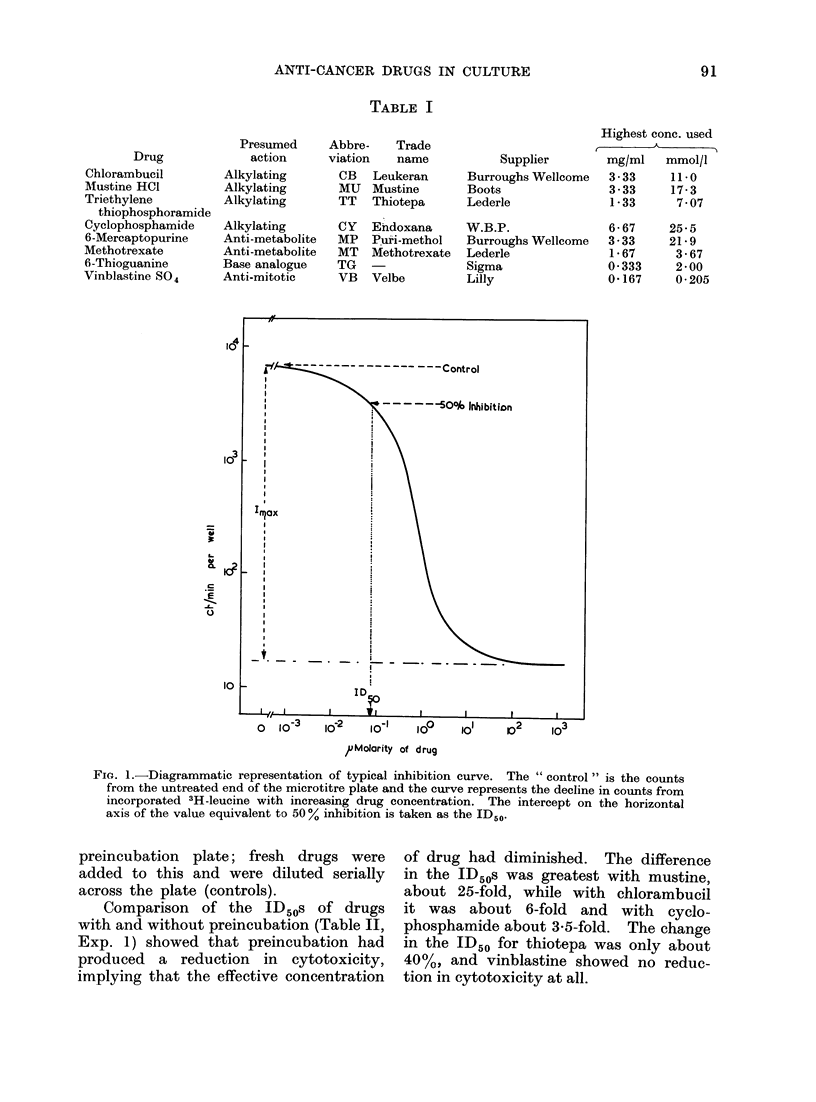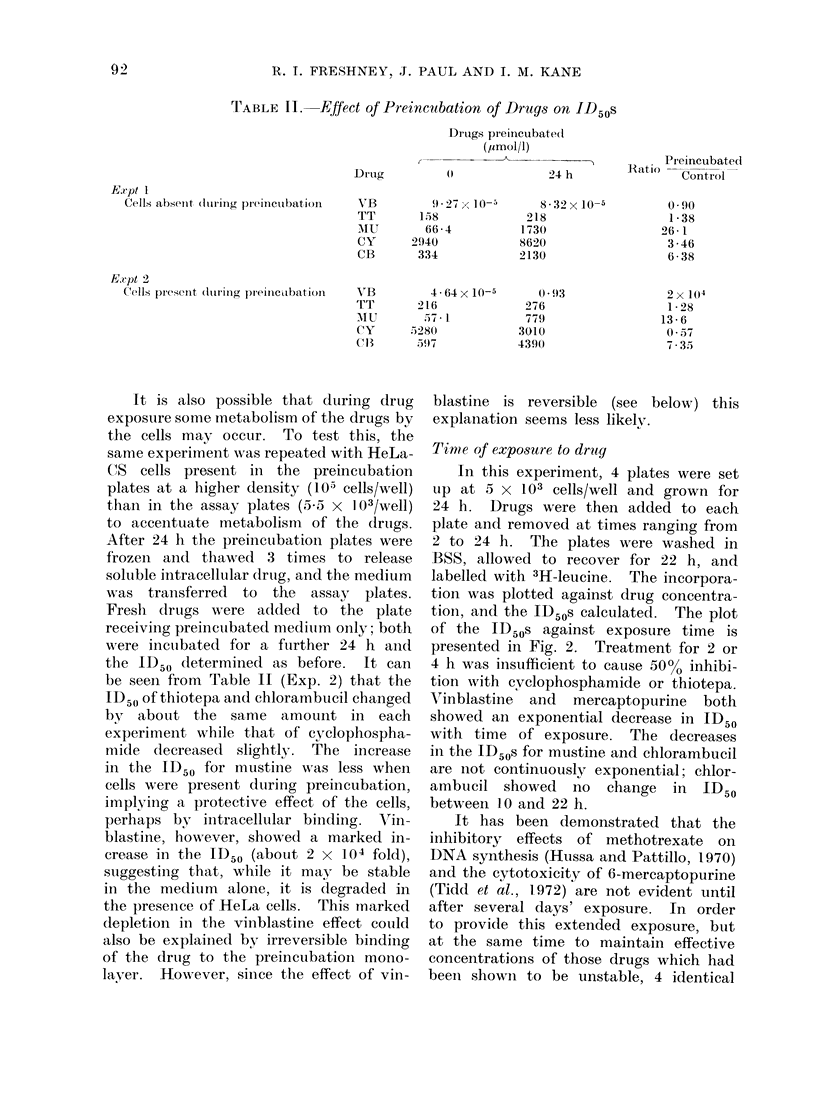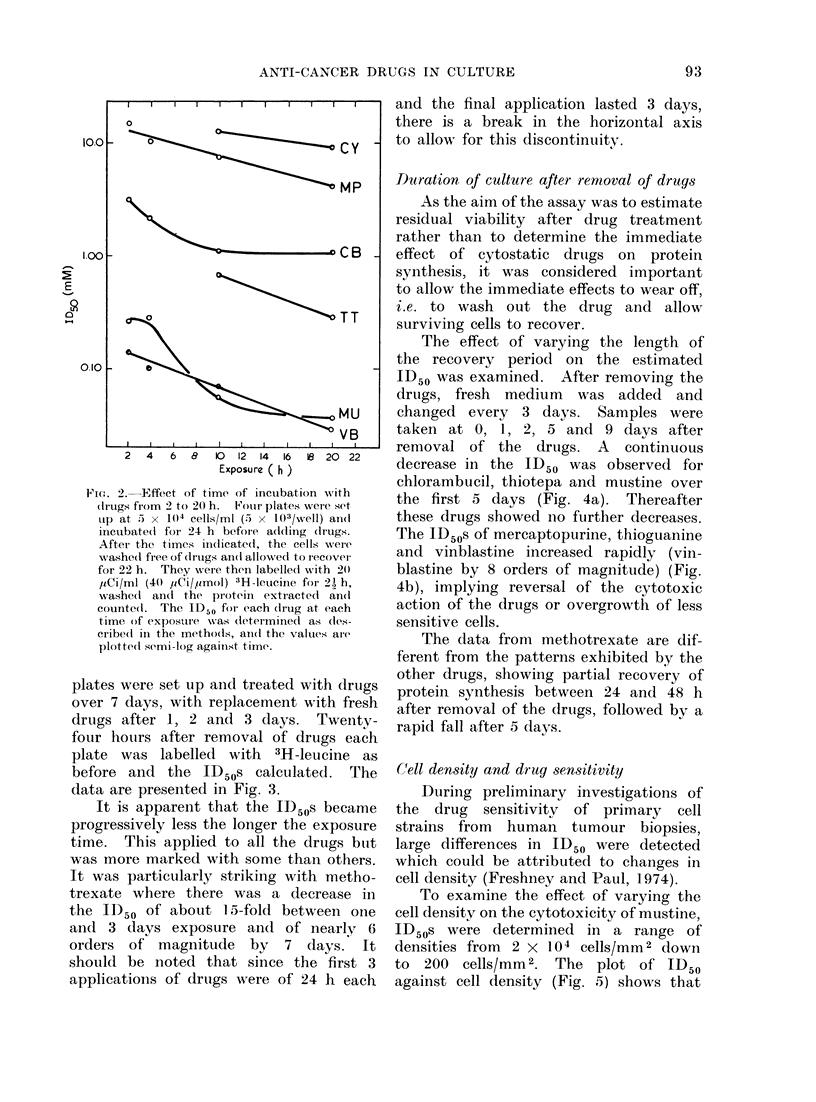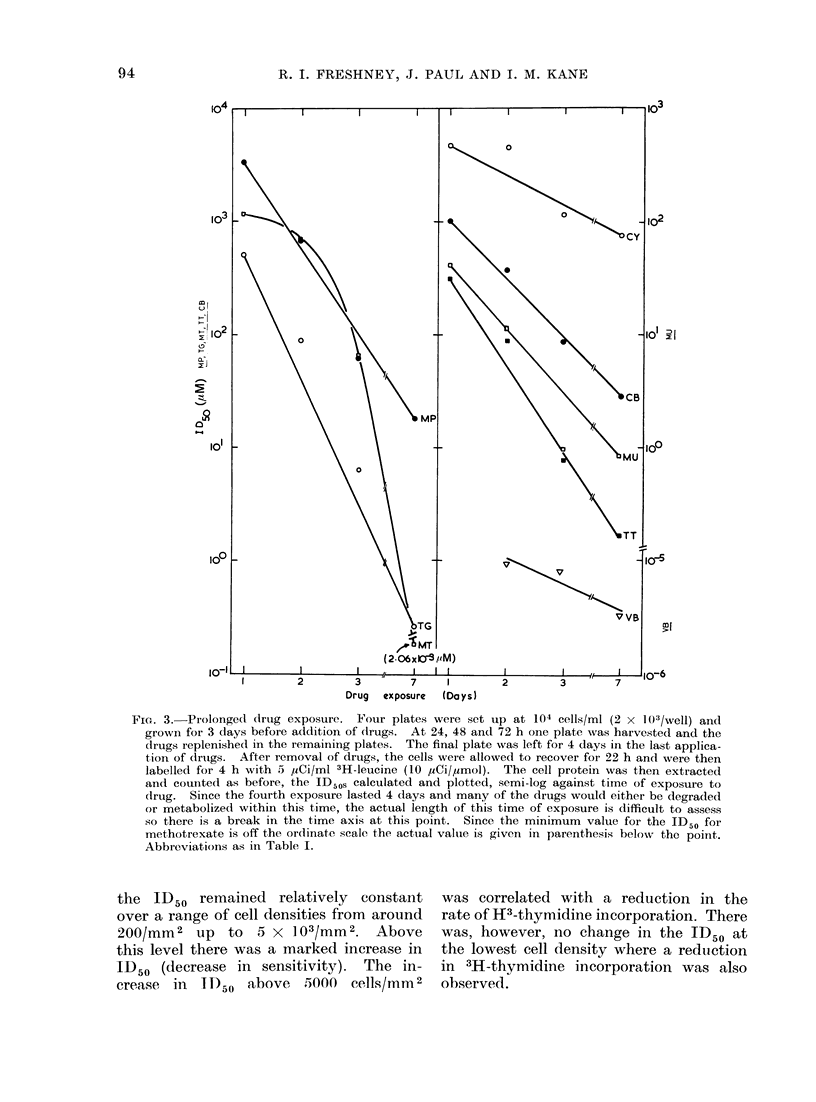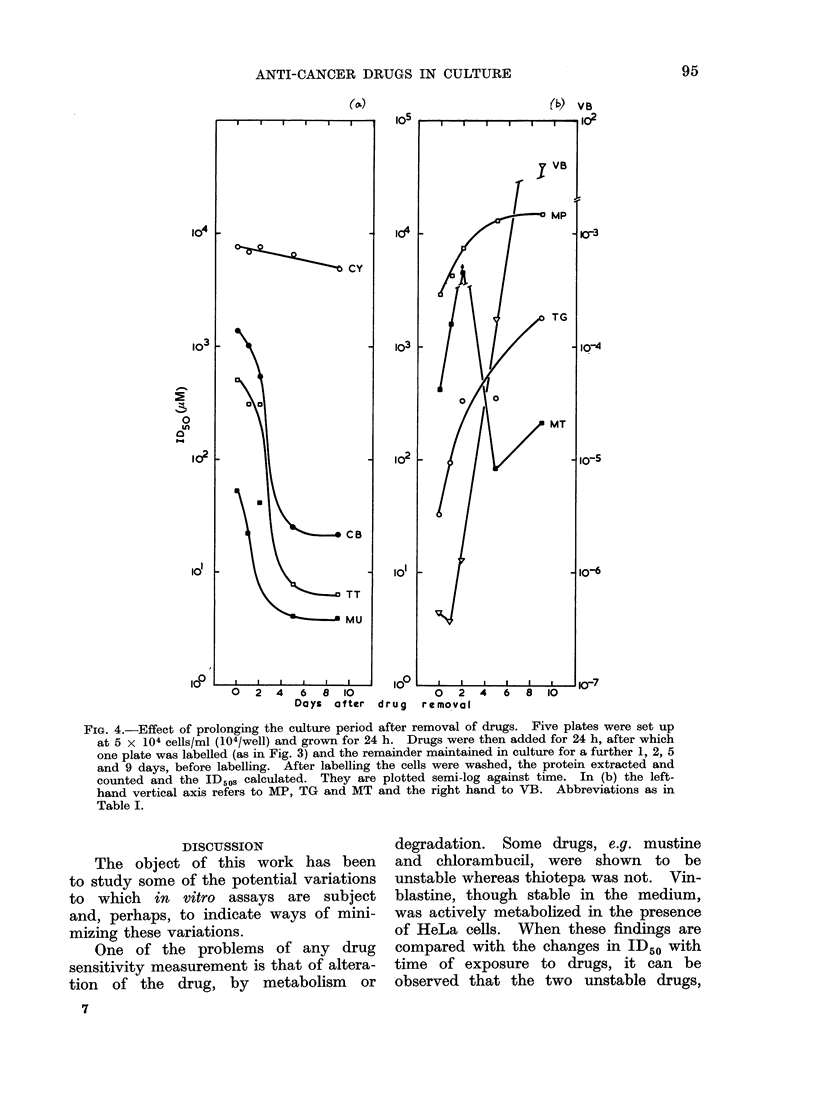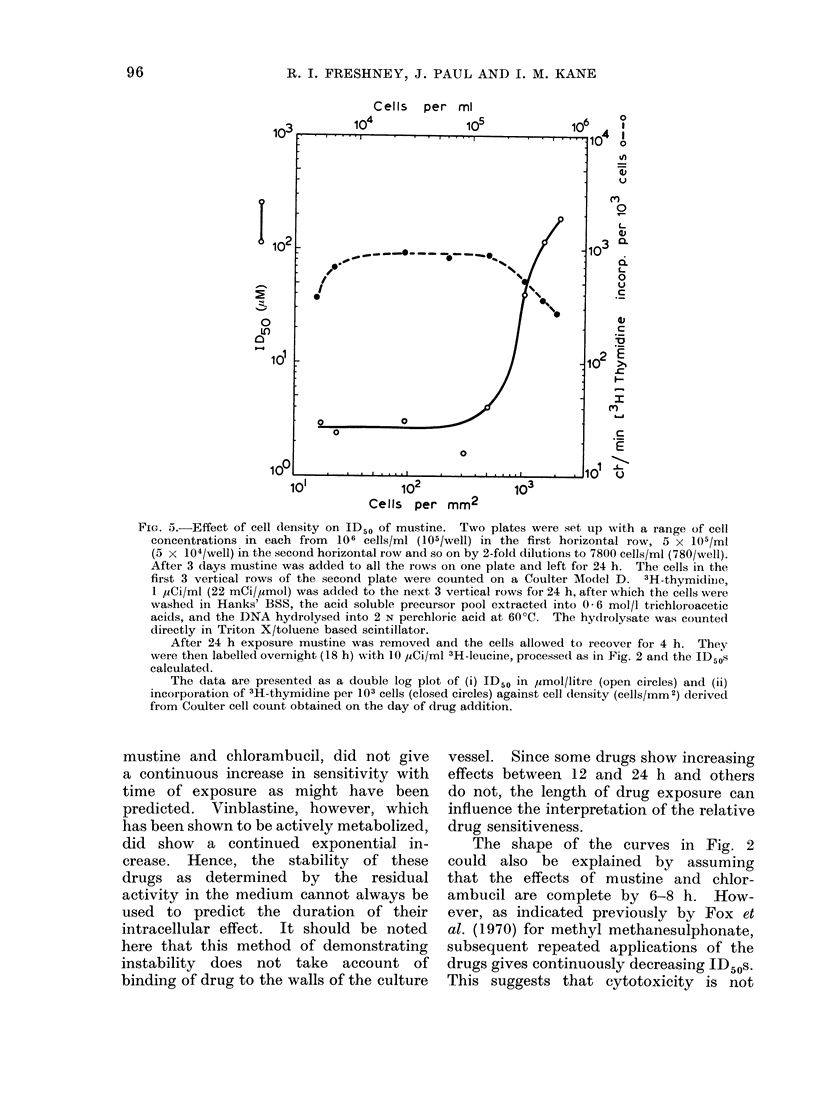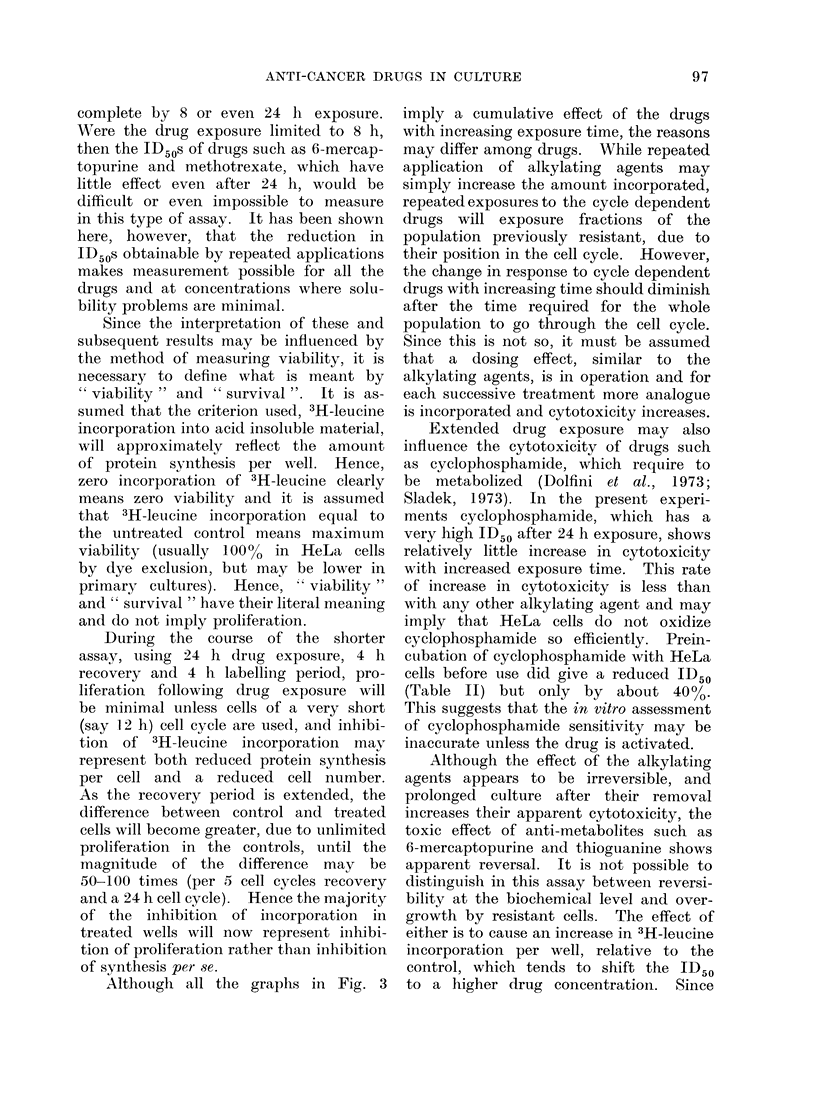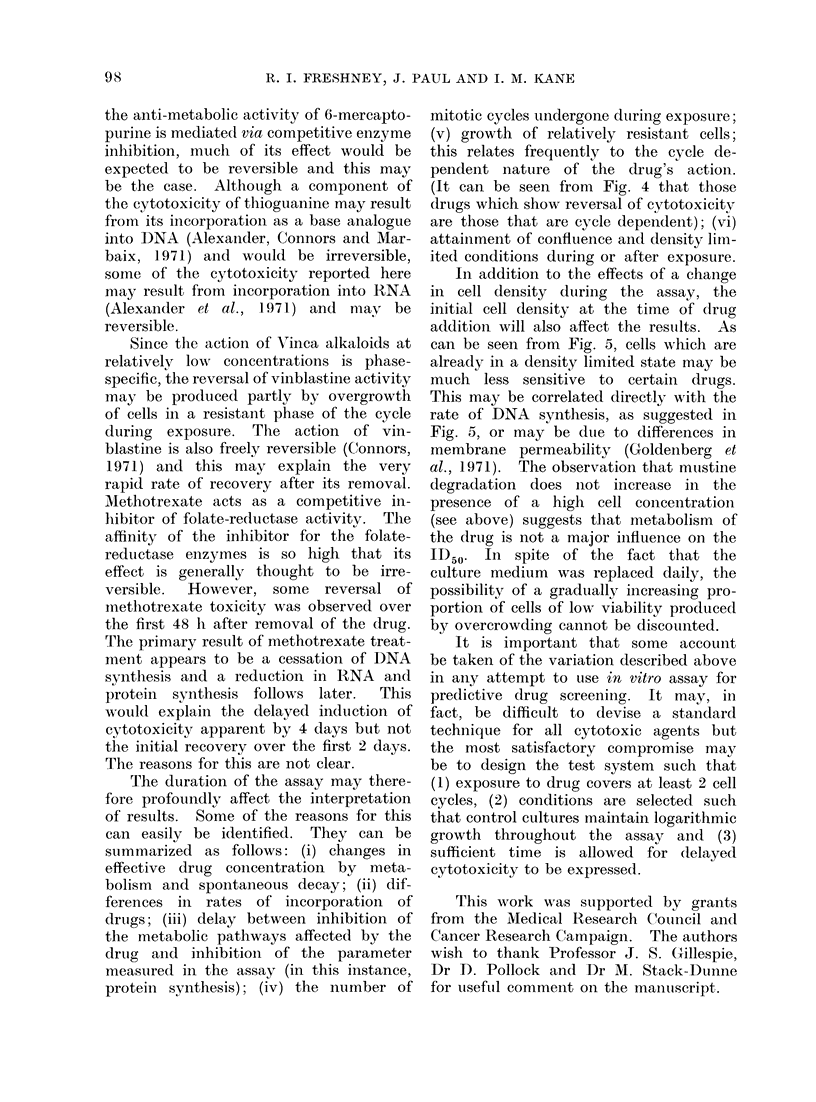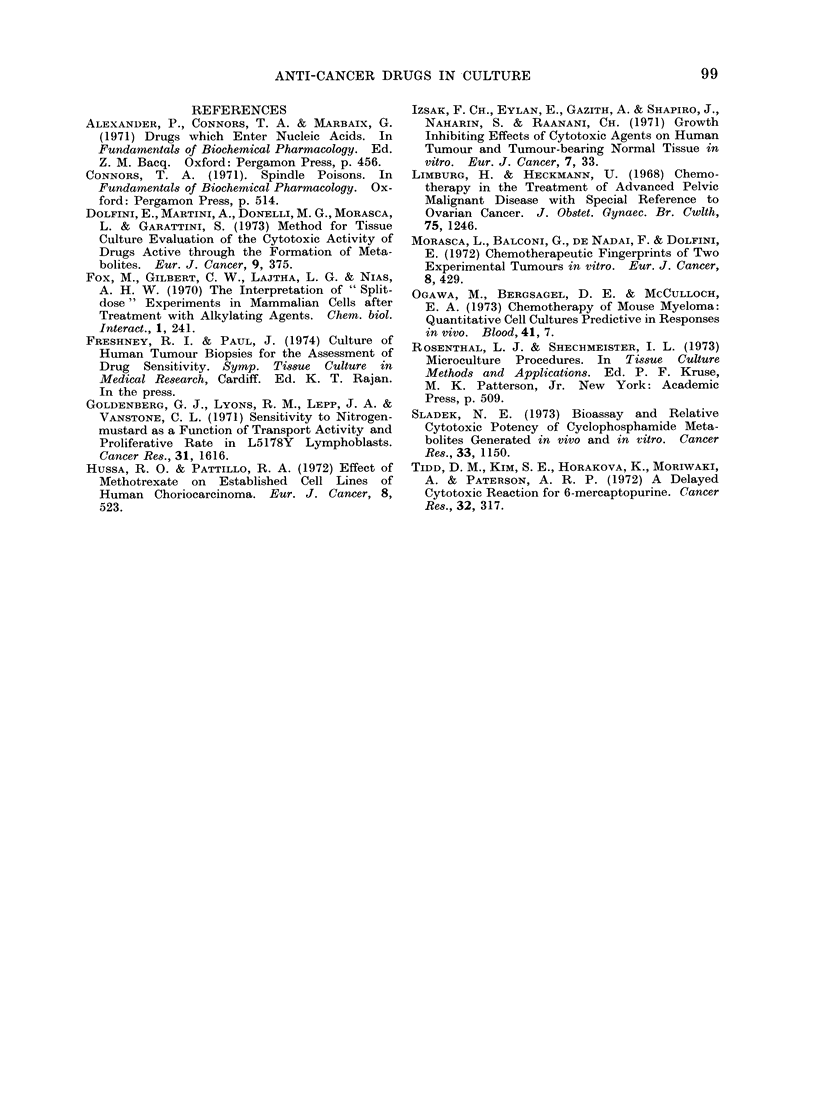# Assay of anti-cancer drugs in tissue culture: conditions affecting their ability to incorporate 3H-leucine after drug treatment.

**DOI:** 10.1038/bjc.1975.11

**Published:** 1975-01

**Authors:** R. I. Freshney, J. Paul, I. M. Kane

## Abstract

An attempt has been made to construct an assay potentially suitable for use with primary cultures of human tumours to measure the survival of exponentially growing monolayer cultures after exposure to anti-neoplastic drugs. Cell survival was assessed using their protein synthetic capacity after removal of drugs. HeLa cells were employed to avoid the ingerent variability and heterogeneity of primary cultures from human tumours, and an assay has been constructed using microtitration trays to provide large numbers of replicate cultures without the requirement of a large number cells. An increase in the duration of the exposure to drug increased sensitivity in nearly all cases examined. Similarly, an increase in the period of culture following drug removal produced increased sensitivity to alkylating agents but allowed recovery from exposure to certain cycle-dependent drugs. Some of the drugs used were shown to be unstable under culture conditions and vinblastine was actively metabolized, although this instability was not necessarily reflected in the time course of the drug's effect. Mustine sensitivity was shown to be reduced by an increase in cell density at a level where density limitation of 3H-thymidine incorporation becomes apparent. These variations and possible methods of minimizing their effects are discussed.


*Br. J. Cancer* (1975) **31**, 89

### ASSAY OF ANTI-CANCER DRUGS IN TISSUE CULTURE; CONDITIONS AFFECTING THEIR ABILITY TO INCORPORATE $^3$H-LEUCINE AFTER DRUG TREATMENT

R. I. FRESHNEY, J. PAUL AND I. M. KANE

*From the Beatson Institute for Cancer Research, 132 Hill Street, Glasgow G3 6UD*

Received 21 August 1974. Accepted 18 September 1974

**Summary.—An attempt has been made to construct an assay potentially suitable for use with primary cultures of human tumours to measure the survival of exponentially growing monolayer cultures after exposure to anti-neoplastic drugs. Cell survival was assessed using their protein synthetic capacity after removal of drugs. HeLa cells were employed to avoid the inherent variability and heterogeneity of primary cultures from human tumours, and an assay has been constructed using microtitration trays to provide large numbers of replicate cultures without the requirement of a large number of cells. An increase in the duration of the exposure to drug increased sensitivity in nearly all cases examined. Similarly, an increase in the period of culture following drug removal produced increased sensitivity to alkylating agents but allowed recovery from exposure to certain cycle-dependent drugs.**

**Some of the drugs used were shown to be unstable under culture conditions and vinblastine was actively metabolized, although this instability was not necessarily reflected in the time course of the drug's effect. Mustine sensitivity was shown to be reduced by an increase in cell density at a level where density limitation of $^3$H-thymidine incorporation becomes apparent.**

**These variations and possible methods of minimizing their effects are discussed.**

CYTOTOXICITY of anti-neoplastic drugs *in vitro* has been used to assess their likely relative potencies in clinical treatment (Limburg and Heckman, 1968; Izsak *et al.*, 1971), and in animal experiments (Morasca *et al.*, 1972; Ogawa, Bergsagel and McCulloch, 1973). There are numerous possible sources of error in this type of screening, including variations in the proportions of different cell types in tissue samples and difficulty in the selection of assay conditions which will give an accurate indication of the sensitivity of the cells to the drugs tested. The relative *in vitro* colony forming ability of treated and untreated cell samples is the most commonly used method of assessing drug sensitivity. Although this method has considerable advantages, it is not readily applied to primary cultures of human tumours due to their very low cloning efficiency and the slow growth rate of the clones.

The adoption of an assay using monolayer cultures, based on their proliferation ability alone, requires extensive cell counting and would become extremely unwieldy where large numbers of samples are used. The metabolic inhibition test, as used in virus assay (Rosenthal and Shechmeister, 1973) is quicker and capable of handling very large numbers of samples. The major difference in this type of test is that metabolic activity is measured rather than proliferation. Protein synthesis was selected in this case as a fundamental metabolic process without which the cell will not survive. $^3$H-

leucine incorporation was used as an index of protein synthesis. As leucine is an essential amino acid, it is unlikely that protein synthesis could continue for long, unimpaired, without leucine incorporation. Hence the absence of protein synthesis, particularly several hours after removal of drug, will almost certainly correlate with cell death.

The relative importance of technical aspects of the assay procedure was suggested by some variations in preliminary results with cultures from human tumour biopsies (Freshney and Paul, 1974). Because of the relatively slow growth rates and heterogeneity of such cultures, we have undertaken the following experiments with HeLa cells in an attempt to identify the most likely sources of variation affecting the validity of this type of assay.

### MATERIALS AND METHODS

*Cell culture.*—HeLa-CS and HeLa-$S_3$ (Fig. 5 only) cells (Flow Laboratories, Irvine) were grown in Ham's F12 medium supplemented with Eagle's MEM amino acids (Flow Laboratories, Irvine), 20% foetal bovine serum (Biocult Laboratories, Paisley) and 50 u/ml benzylpenicillin. Although more than adequate for HeLa cells, this medium was selected to be consistent with parallel experiments with primary tumour cultures which require a highly enriched medium.

*Drug sensitivity experiments.*—The following procedure was used for all experiments with individual modifications as noted in the figure legends: Monolayer cultures were trypsinized and transferred to microtitre trays (Flow Laboratories), each tray having 8 horizontal rows of 12 wells. After 1–3 days, drugs were added to the extreme left-hand wells and serially diluted across the plate. The last 2 wells in each row were left free of drug to act as controls. After 24 h the drug solutions were removed by suction, the monolayer washed with Hanks' balanced salt solution (BSS) and fresh growth medium added for a further 4–24 h. This was then replaced with growth medium containing 5 $\mu$Ci/ml L-leucine-4, 5-$^3$H (10 $\mu$Ci/$\mu$mol final specific activity) (Radiochemical Centre, Amersham) and incubation continued for a further 4–24 h. When the length of the labelling period was varied it was shown that the incorporation of $^3$H leucine increased proportionally with time over 2–20 h. The plates were then washed in BSS, fixed in methanol and dried. Acid-soluble precursors were extracted with two 10-min washes of ice-cold 0·6 mol/l trichloroacetic acid, the trichloroacetic acid washed off with cold distilled water and the residual cellular material dissolved in 1 N NaOH. The samples were acidified with excess 1·1 N HCl and the radioactivity determined by scintillation counting.

The drugs used were as in Table I.

*Presentation of data.*—When the $^3$H-leucine incorporation per well is plotted against the drug concentration a sigmoid curve is obtained, the typical form of which is represented diagrammatically in Fig. 1. In most cases where the bottom end of the curve flattens out, $^3$H-leucine incorporation is close to the background for the assay (10–20 ct/min per well). The maximum inhibition ($I_{max}$), and the drug concentration at 50% inhibition ($ID_{50}$) may be determined from the curve. The $ID_{50}$ values are then available for plotting against a third variable such as cell density, time of exposure to drug etc.

In later experiments, the derivation of these values has been automated using a punched tape output from the scintillation counter and analysing the tape on a Wang desk-top computer. The authors are indebted to Dr Bryan Young of this Institute for the provision of a programme for this operation.

### RESULTS

### *Stability and metabolism of drugs during incubation*

To test for degradation of drugs 4 microtitre plates were set up, 2 containing cells at $5 \times 10^3$/well ($5 \times 10^4$/ml) and no drugs (assay plates), one containing serial dilutions of 5 drugs but no cells (drug preincubation plate), and one containing medium only (medium preincubation plate). After 24 h the medium from one assay plate was discarded and replaced with the medium from the drug preincubation plate. The medium from the second assay plate was removed and replaced with medium from the medium

TABLE I

| Drug | Presumed action | Abbreviation | Trade name | Supplier | Highest conc. used mg/ml | Highest conc. used mmol/l |
|---|---|---|---|---|---|---|
| Chlorambucil | Alkylating | CB | Leukeran | Burroughs Wellcome | 3·33 | 11·0 |
| Mustine HCl | Alkylating | MU | Mustine | Boots | 3·33 | 17·3 |
| Triethylene thiophosphoramide | Alkylating | TT | Thiotepa | Lederle | 1·33 | 7·07 |
| Cyclophosphamide | Alkylating | CY | Endoxana | W.B.P. | 6·67 | 25·5 |
| 6-Mercaptopurine | Anti-metabolite | MP | Puri-methol | Burroughs Wellcome | 3·33 | 21·9 |
| Methotrexate | Anti-metabolite | MT | Methotrexate | Lederle | 1·67 | 3·67 |
| 6-Thioguanine | Base analogue | TG | — | Sigma | 0·333 | 2·00 |
| Vinblastine $SO_4$ | Anti-mitotic | VB | Velbe | Lilly | 0·167 | 0·205 |

FIG. 1.—Diagrammatic representation of typical inhibition curve. The " control " is the counts from the untreated end of the microtitre plate and the curve represents the decline in counts from incorporated $^3$H-leucine with increasing drug concentration. The intercept on the horizontal axis of the value equivalent to 50% inhibition is taken as the $ID_{50}$.

preincubation plate; fresh drugs were added to this and were diluted serially across the plate (controls).

Comparison of the $ID_{50}$s of drugs with and without preincubation (Table II, Exp. 1) showed that preincubation had produced a reduction in cytotoxicity, implying that the effective concentration of drug had diminished. The difference in the $ID_{50}$s was greatest with mustine, about 25-fold, while with chlorambucil it was about 6-fold and with cyclophosphamide about 3·5-fold. The change in the $ID_{50}$ for thiotepa was only about 40%, and vinblastine showed no reduction in cytotoxicity at all.

TABLE II.—*Effect of Preincubation of Drugs on $ID_{50}$s*

| | Drug | Drugs preincubated (μmol/l) 0 | 24 h | Ratio Preincubated/Control |
|---|---|---|---|---|
| *Expt* 1 | | | | |
| Cells absent during preincubation | VB | $9 \cdot 27 \times 10^{-5}$ | $8 \cdot 32 \times 10^{-5}$ | 0·90 |
| | TT | 158 | 218 | 1·38 |
| | MU | 66·4 | 1730 | 26·1 |
| | CY | 2940 | 8620 | 3·46 |
| | CB | 334 | 2130 | 6·38 |
| *Expt* 2 | | | | |
| Cells present during preincubation | VB | $4 \cdot 64 \times 10^{-5}$ | 0·93 | $2 \times 10^{4}$ |
| | TT | 216 | 276 | 1·28 |
| | MU | 57·1 | 779 | 13·6 |
| | CY | 5280 | 3010 | 0·57 |
| | CB | 597 | 4390 | 7·35 |

It is also possible that during drug exposure some metabolism of the drugs by the cells may occur. To test this, the same experiment was repeated with HeLa-CS cells present in the preincubation plates at a higher density ($10^5$ cells/well) than in the assay plates ($5{\cdot}5 \times 10^3$/well) to accentuate metabolism of the drugs. After 24 h the preincubation plates were frozen and thawed 3 times to release soluble intracellular drug, and the medium was transferred to the assay plates. Fresh drugs were added to the plate receiving preincubated medium only; both were incubated for a further 24 h and the $ID_{50}$ determined as before. It can be seen from Table II (Exp. 2) that the $ID_{50}$ of thiotepa and chlorambucil changed by about the same amount in each experiment while that of cyclophosphamide decreased slightly. The increase in the $ID_{50}$ for mustine was less when cells were present during preincubation, implying a protective effect of the cells, perhaps by intracellular binding. Vinblastine, however, showed a marked increase in the $ID_{50}$ (about $2 \times 10^4$ fold), suggesting that, while it may be stable in the medium alone, it is degraded in the presence of HeLa cells. This marked depletion in the vinblastine effect could also be explained by irreversible binding of the drug to the preincubation monolayer. However, since the effect of vinblastine is reversible (see below) this explanation seems less likely.

*Time of exposure to drug*

In this experiment, 4 plates were set up at $5 \times 10^3$ cells/well and grown for 24 h. Drugs were then added to each plate and removed at times ranging from 2 to 24 h. The plates were washed in BSS, allowed to recover for 22 h, and labelled with $^3$H-leucine. The incorporation was plotted against drug concentration, and the $ID_{50}$s calculated. The plot of the $ID_{50}$s against exposure time is presented in Fig. 2. Treatment for 2 or 4 h was insufficient to cause 50% inhibition with cyclophosphamide or thiotepa. Vinblastine and mercaptopurine both showed an exponential decrease in $ID_{50}$ with time of exposure. The decreases in the $ID_{50}$s for mustine and chlorambucil are not continuously exponential; chlorambucil showed no change in $ID_{50}$ between 10 and 22 h.

It has been demonstrated that the inhibitory effects of methotrexate on DNA synthesis (Hussa and Pattillo, 1970) and the cytotoxicity of 6-mercaptopurine (Tidd *et al.*, 1972) are not evident until after several days' exposure. In order to provide this extended exposure, but at the same time to maintain effective concentrations of those drugs which had been shown to be unstable, 4 identical

FIG. 2.—Effect of time of incubation with drugs from 2 to 20 h. Four plates were set up at $5 \times 10^4$ cells/ml ($5 \times 10^3$/well) and incubated for 24 h before adding drugs. After the times indicated, the cells were washed free of drugs and allowed to recover for 22 h. They were then labelled with 20 $\mu$Ci/ml (40 $\mu$Ci/$\mu$mol) $^3$H-leucine for $2\frac{1}{2}$ h, washed and the protein extracted and counted. The $ID_{50}$ for each drug at each time of exposure was determined as described in the methods, and the values are plotted semi-log against time.

plates were set up and treated with drugs over 7 days, with replacement with fresh drugs after 1, 2 and 3 days. Twenty-four hours after removal of drugs each plate was labelled with $^3$H-leucine as before and the $ID_{50}$s calculated. The data are presented in Fig. 3.

It is apparent that the $ID_{50}$s became progressively less the longer the exposure time. This applied to all the drugs but was more marked with some than others. It was particularly striking with methotrexate where there was a decrease in the $ID_{50}$ of about 15-fold between one and 3 days exposure and of nearly 6 orders of magnitude by 7 days. It should be noted that since the first 3 applications of drugs were of 24 h each and the final application lasted 3 days, there is a break in the horizontal axis to allow for this discontinuity.

*Duration of culture after removal of drugs*

As the aim of the assay was to estimate residual viability after drug treatment rather than to determine the immediate effect of cytostatic drugs on protein synthesis, it was considered important to allow the immediate effects to wear off, *i.e.* to wash out the drug and allow surviving cells to recover.

The effect of varying the length of the recovery period on the estimated $ID_{50}$ was examined. After removing the drugs, fresh medium was added and changed every 3 days. Samples were taken at 0, 1, 2, 5 and 9 days after removal of the drugs. A continuous decrease in the $ID_{50}$ was observed for chlorambucil, thiotepa and mustine over the first 5 days (Fig. 4a). Thereafter these drugs showed no further decreases. The $ID_{50}$s of mercaptopurine, thioguanine and vinblastine increased rapidly (vinblastine by 8 orders of magnitude) (Fig. 4b), implying reversal of the cytotoxic action of the drugs or overgrowth of less sensitive cells.

The data from methotrexate are different from the patterns exhibited by the other drugs, showing partial recovery of protein synthesis between 24 and 48 h after removal of the drugs, followed by a rapid fall after 5 days.

*Cell density and drug sensitivity*

During preliminary investigations of the drug sensitivity of primary cell strains from human tumour biopsies, large differences in $ID_{50}$ were detected which could be attributed to changes in cell density (Freshney and Paul, 1974).

To examine the effect of varying the cell density on the cytotoxicity of mustine, $ID_{50}$s were determined in a range of densities from $2 \times 10^4$ cells/mm$^2$ down to 200 cells/mm$^2$. The plot of $ID_{50}$ against cell density (Fig. 5) shows that

FIG. 3.—Prolonged drug exposure. Four plates were set up at $10^4$ cells/ml ($2 \times 10^3$/well) and grown for 3 days before addition of drugs. At 24, 48 and 72 h one plate was harvested and the drugs replenished in the remaining plates. The final plate was left for 4 days in the last application of drugs. After removal of drugs, the cells were allowed to recover for 22 h and were then labelled for 4 h with 5 μCi/ml $^3$H-leucine (10 μCi/μmol). The cell protein was then extracted and counted as before, the $ID_{50}$s calculated and plotted, semi-log against time of exposure to drug. Since the fourth exposure lasted 4 days and many of the drugs would either be degraded or metabolized within this time, the actual length of this time of exposure is difficult to assess so there is a break in the time axis at this point. Since the minimum value for the $ID_{50}$ for methotrexate is off the ordinate scale the actual value is given in parenthesis below the point. Abbreviations as in Table I.

the $ID_{50}$ remained relatively constant over a range of cell densities from around 200/mm$^2$ up to $5 \times 10^3$/mm$^2$. Above this level there was a marked increase in $ID_{50}$ (decrease in sensitivity). The increase in $ID_{50}$ above 5000 cells/mm$^2$ was correlated with a reduction in the rate of $H^3$-thymidine incorporation. There was, however, no change in the $ID_{50}$ at the lowest cell density where a reduction in $^3$H-thymidine incorporation was also observed.

FIG. 4.—Effect of prolonging the culture period after removal of drugs. Five plates were set up at 5 × $10^4$ cells/ml ($10^4$/well) and grown for 24 h. Drugs were then added for 24 h, after which one plate was labelled (as in Fig. 3) and the remainder maintained in culture for a further 1, 2, 5 and 9 days, before labelling. After labelling the cells were washed, the protein extracted and counted and the $ID_{50}$s calculated. They are plotted semi-log against time. In (b) the left-hand vertical axis refers to MP, TG and MT and the right hand to VB. Abbreviations as in Table I.

### DISCUSSION

The object of this work has been to study some of the potential variations to which *in vitro* assays are subject and, perhaps, to indicate ways of minimizing these variations.

One of the problems of any drug sensitivity measurement is that of alteration of the drug, by metabolism or degradation. Some drugs, *e.g.* mustine and chlorambucil, were shown to be unstable whereas thiotepa was not. Vinblastine, though stable in the medium, was actively metabolized in the presence of HeLa cells. When these findings are compared with the changes in $ID_{50}$ with time of exposure to drugs, it can be observed that the two unstable drugs,

FIG. 5.—Effect of cell density on $ID_{50}$ of mustine. Two plates were set up with a range of cell concentrations in each from $10^6$ cells/ml ($10^5$/well) in the first horizontal row, $5 \times 10^5$/ml ($5 \times 10^4$/well) in the second horizontal row and so on by 2-fold dilutions to 7800 cells/ml (780/well). After 3 days mustine was added to all the rows on one plate and left for 24 h. The cells in the first 3 vertical rows of the second plate were counted on a Coulter Model D. $^3$H-thymidine, 1 μCi/ml (22 mCi/μmol) was added to the next 3 vertical rows for 24 h, after which the cells were washed in Hanks' BSS, the acid soluble precursor pool extracted into 0·6 mol/l trichloroacetic acids, and the DNA hydrolysed into 2 N perchloric acid at 60°C. The hydrolysate was counted directly in Triton X/toluene based scintillator.

After 24 h exposure mustine was removed and the cells allowed to recover for 4 h. They were then labelled overnight (18 h) with 10 μCi/ml $^3$H-leucine, processed as in Fig. 2 and the $ID_{50}$s calculated.

The data are presented as a double log plot of (i) $ID_{50}$ in μmol/litre (open circles) and (ii) incorporation of $^3$H-thymidine per $10^3$ cells (closed circles) against cell density (cells/mm$^2$) derived from Coulter cell count obtained on the day of drug addition.

mustine and chlorambucil, did not give a continuous increase in sensitivity with time of exposure as might have been predicted. Vinblastine, however, which has been shown to be actively metabolized, did show a continued exponential increase. Hence, the stability of these drugs as determined by the residual activity in the medium cannot always be used to predict the duration of their intracellular effect. It should be noted here that this method of demonstrating instability does not take account of binding of drug to the walls of the culture vessel. Since some drugs show increasing effects between 12 and 24 h and others do not, the length of drug exposure can influence the interpretation of the relative drug sensitiveness.

The shape of the curves in Fig. 2 could also be explained by assuming that the effects of mustine and chlorambucil are complete by 6–8 h. However, as indicated previously by Fox *et al.* (1970) for methyl methanesulphonate, subsequent repeated applications of the drugs gives continuously decreasing $ID_{50}$s. This suggests that cytotoxicity is not complete by 8 or even 24 h exposure. Were the drug exposure limited to 8 h, then the $ID_{50}$s of drugs such as 6-mercaptopurine and methotrexate, which have little effect even after 24 h, would be difficult or even impossible to measure in this type of assay. It has been shown here, however, that the reduction in $ID_{50}$s obtainable by repeated applications makes measurement possible for all the drugs and at concentrations where solubility problems are minimal.

Since the interpretation of these and subsequent results may be influenced by the method of measuring viability, it is necessary to define what is meant by " viability " and " survival ". It is assumed that the criterion used, $^3$H-leucine incorporation into acid insoluble material, will approximately reflect the amount of protein synthesis per well. Hence, zero incorporation of $^3$H-leucine clearly means zero viability and it is assumed that $^3$H-leucine incorporation equal to the untreated control means maximum viability (usually 100% in HeLa cells by dye exclusion, but may be lower in primary cultures). Hence, " viability " and " survival " have their literal meaning and do not imply proliferation.

During the course of the shorter assay, using 24 h drug exposure, 4 h recovery and 4 h labelling period, proliferation following drug exposure will be minimal unless cells of a very short (say 12 h) cell cycle are used, and inhibition of $^3$H-leucine incorporation may represent both reduced protein synthesis per cell and a reduced cell number. As the recovery period is extended, the difference between control and treated cells will become greater, due to unlimited proliferation in the controls, until the magnitude of the difference may be 50–100 times (per 5 cell cycles recovery and a 24 h cell cycle). Hence the majority of the inhibition of incorporation in treated wells will now represent inhibition of proliferation rather than inhibition of synthesis *per se*.

Although all the graphs in Fig. 3 imply a cumulative effect of the drugs with increasing exposure time, the reasons may differ among drugs. While repeated application of alkylating agents may simply increase the amount incorporated, repeated exposures to the cycle dependent drugs will exposure fractions of the population previously resistant, due to their position in the cell cycle. However, the change in response to cycle dependent drugs with increasing time should diminish after the time required for the whole population to go through the cell cycle. Since this is not so, it must be assumed that a dosing effect, similar to the alkylating agents, is in operation and for each successive treatment more analogue is incorporated and cytotoxicity increases.

Extended drug exposure may also influence the cytotoxicity of drugs such as cyclophosphamide, which require to be metabolized (Dolfini *et al.*, 1973; Sladek, 1973). In the present experiments cyclophosphamide, which has a very high $ID_{50}$ after 24 h exposure, shows relatively little increase in cytotoxicity with increased exposure time. This rate of increase in cytotoxicity is less than with any other alkylating agent and may imply that HeLa cells do not oxidize cyclophosphamide so efficiently. Preincubation of cyclophosphamide with HeLa cells before use did give a reduced $ID_{50}$ (Table II) but only by about 40%. This suggests that the *in vitro* assessment of cyclophosphamide sensitivity may be inaccurate unless the drug is activated.

Although the effect of the alkylating agents appears to be irreversible, and prolonged culture after their removal increases their apparent cytotoxicity, the toxic effect of anti-metabolites such as 6-mercaptopurine and thioguanine shows apparent reversal. It is not possible to distinguish in this assay between reversibility at the biochemical level and overgrowth by resistant cells. The effect of either is to cause an increase in $^3$H-leucine incorporation per well, relative to the control, which tends to shift the $ID_{50}$ to a higher drug concentration. Since the anti-metabolic activity of 6-mercaptopurine is mediated *via* competitive enzyme inhibition, much of its effect would be expected to be reversible and this may be the case. Although a component of the cytotoxicity of thioguanine may result from its incorporation as a base analogue into DNA (Alexander, Connors and Marbaix, 1971) and would be irreversible, some of the cytotoxicity reported here may result from incorporation into RNA (Alexander *et al.*, 1971) and may be reversible.

Since the action of Vinca alkaloids at relatively low concentrations is phase-specific, the reversal of vinblastine activity may be produced partly by overgrowth of cells in a resistant phase of the cycle during exposure. The action of vinblastine is also freely reversible (Connors, 1971) and this may explain the very rapid rate of recovery after its removal. Methotrexate acts as a competitive inhibitor of folate-reductase activity. The affinity of the inhibitor for the folate-reductase enzymes is so high that its effect is generally thought to be irreversible. However, some reversal of methotrexate toxicity was observed over the first 48 h after removal of the drug. The primary result of methotrexate treatment appears to be a cessation of DNA synthesis and a reduction in RNA and protein synthesis follows later. This would explain the delayed induction of cytotoxicity apparent by 4 days but not the initial recovery over the first 2 days. The reasons for this are not clear.

The duration of the assay may therefore profoundly affect the interpretation of results. Some of the reasons for this can easily be identified. They can be summarized as follows: (i) changes in effective drug concentration by metabolism and spontaneous decay; (ii) differences in rates of incorporation of drugs; (iii) delay between inhibition of the metabolic pathways affected by the drug and inhibition of the parameter measured in the assay (in this instance, protein synthesis); (iv) the number of mitotic cycles undergone during exposure; (v) growth of relatively resistant cells; this relates frequently to the cycle dependent nature of the drug's action. (It can be seen from Fig. 4 that those drugs which show reversal of cytotoxicity are those that are cycle dependent); (vi) attainment of confluence and density limited conditions during or after exposure.

In addition to the effects of a change in cell density during the assay, the initial cell density at the time of drug addition will also affect the results. As can be seen from Fig. 5, cells which are already in a density limited state may be much less sensitive to certain drugs. This may be correlated directly with the rate of DNA synthesis, as suggested in Fig. 5, or may be due to differences in membrane permeability (Goldenberg *et al.*, 1971). The observation that mustine degradation does not increase in the presence of a high cell concentration (see above) suggests that metabolism of the drug is not a major influence on the $ID_{50}$. In spite of the fact that the culture medium was replaced daily, the possibility of a gradually increasing proportion of cells of low viability produced by overcrowding cannot be discounted.

It is important that some account be taken of the variation described above in any attempt to use *in vitro* assay for predictive drug screening. It may, in fact, be difficult to devise a standard technique for all cytotoxic agents but the most satisfactory compromise may be to design the test system such that (1) exposure to drug covers at least 2 cell cycles, (2) conditions are selected such that control cultures maintain logarithmic growth throughout the assay and (3) sufficient time is allowed for delayed cytotoxicity to be expressed.

This work was supported by grants from the Medical Research Council and Cancer Research Campaign. The authors wish to thank Professor J. S. Gillespie, Dr D. Pollock and Dr M. Stack-Dunne for useful comment on the manuscript.

### REFERENCES

ALEXANDER, P., CONNORS, T. A. & MARBAIX, G. (1971) Drugs which Enter Nucleic Acids. In *Fundamentals of Biochemical Pharmacology*. Ed. Z. M. Bacq. Oxford: Pergamon Press, p. 456.

CONNORS, T. A. (1971). Spindle Poisons. In *Fundamentals of Biochemical Pharmacology*. Oxford: Pergamon Press, p. 514.

DOLFINI, E., MARTINI, A., DONELLI, M. G., MORASCA, L. & GARATTINI, S. (1973) Method for Tissue Culture Evaluation of the Cytotoxic Activity of Drugs Active through the Formation of Metabolites. *Eur. J. Cancer*, **9**, 375.

FOX, M., GILBERT, C. W., LAJTHA, L. G. & NIAS, A. H. W. (1970) The Interpretation of " Split-dose " Experiments in Mammalian Cells after Treatment with Alkylating Agents. *Chem. biol. Interact.*, **1**, 241.

FRESHNEY, R. I. & PAUL, J. (1974) Culture of Human Tumour Biopsies for the Assessment of Drug Sensitivity. *Symp. Tissue Culture in Medical Research*, Cardiff. Ed. K. T. Rajan. In the press.

GOLDENBERG, G. J., LYONS, R. M., LEPP, J. A. & VANSTONE, C. L. (1971) Sensitivity to Nitrogen-mustard as a Function of Transport Activity and Proliferative Rate in L5178Y Lymphoblasts. *Cancer Res.*, **31**, 1616.

HUSSA, R. O. & PATTILLO, R. A. (1972) Effect of Methotrexate on Established Cell Lines of Human Choriocarcinoma. *Eur. J. Cancer*, **8**, 523.

IZSAK, F. CH., EYLAN, E., GAZITH, A. & SHAPIRO, J., NAHARIN, S. & RAANANI, CH. (1971) Growth Inhibiting Effects of Cytotoxic Agents on Human Tumour and Tumour-bearing Normal Tissue *in vitro*. *Eur. J. Cancer*, **7**, 33.

LIMBURG, H. & HECKMANN, U. (1968) Chemotherapy in the Treatment of Advanced Pelvic Malignant Disease with Special Reference to Ovarian Cancer. *J. Obstet. Gynaec. Br. Cwlth*, **75**, 1246.

MORASCA, L., BALCONI, G., DE NADAI, F. & DOLFINI, E. (1972) Chemotherapeutic Fingerprints of Two Experimental Tumours *in vitro*. *Eur. J. Cancer*, **8**, 429.

OGAWA, M., BERGSAGEL, D. E. & MCCULLOCH, E. A. (1973) Chemotherapy of Mouse Myeloma: Quantitative Cell Cultures Predictive in Responses *in vivo*. *Blood*, **41**, 7.

ROSENTHAL, L. J. & SHECHMEISTER, I. L. (1973) Microculture Procedures. In *Tissue Culture Methods and Applications*. Ed. P. F. Kruse, M. K. Patterson, Jr. New York: Academic Press, p. 509.

SLADEK, N. E. (1973) Bioassay and Relative Cytotoxic Potency of Cyclophosphamide Metabolites Generated *in vivo* and *in vitro*. *Cancer Res.*, **33**, 1150.

TIDD, D. M., KIM, S. E., HORAKOVA, K., MORIWAKI, A. & PATERSON, A. R. P. (1972) A Delayed Cytotoxic Reaction for 6-mercaptopurine. *Cancer Res.*, **32**, 317.